# Supplementary material for: Single-shot laser-driven neutron resonance spectroscopy for temperature profiling
Source: Nat Commun. 2024 Jul 12;15:5365. doi: 10.1038/s41467-024-49142-y (PMC11245602; doi:10.1038/s41467-024-49142-y)
Supplement: Supplementary file 1 — Supplementary Information [file 41467_2024_49142_MOESM1_ESM.pdf]

Supplementary information

Supplementary figure

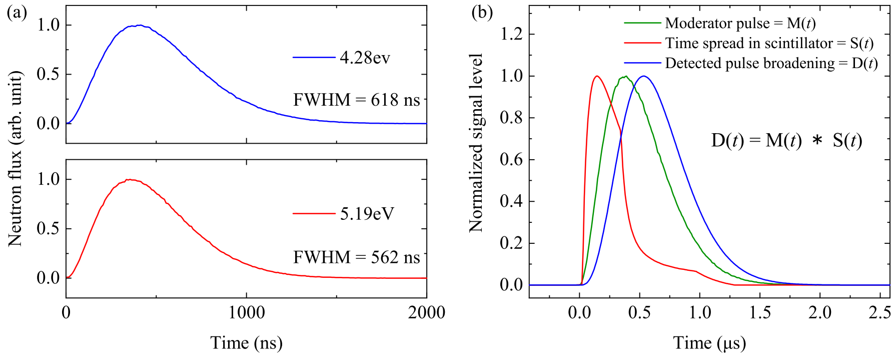

Figure 1: The PHITS simulation of moderated neutron pulse and the evaluated broadening in neutron beamline. (a) Simulated pulse duration for 5.19 eV and 4.28 eV neutrons at the exit of the moderator. (b) Simulated results of pulse broadening at the neutron moderator (green line) and the ^{6}Li glass scintillator (red line). The convolved result at the detector at 1.78 m is shown as a blue line.
